# Human iNKT Cells Modulate Macrophage Survival and Phenotype

**DOI:** 10.3390/biomedicines10071723

**Published:** 2022-07-17

**Authors:** J. Pedro Loureiro, Mariana S. Cruz, Ana P. Cardoso, Maria J. Oliveira, M. Fátima Macedo

**Affiliations:** 1 Cell Activation and Gene Expression Group, Institute for Molecular and Cell Biology (IBMC), Institute for Research and Innovation in Health (i3S), University of Porto, Rua Alfredo Allen 208, 4200-135 Porto, Portugal; josepedro.pereiraloureiro@unibas.ch (J.P.L.); marianascruz@ua.pt (M.S.C.); 2 Experimental Immunology Group, Department of Biomedicine (DBM), University Hospital Basel, University of Basel, Hebelstrasse 20, 4031 Basel, Switzerland; 3 Department of Medical Sciences, University of Aveiro (UA), 3810-193 Aveiro, Portugal; 4 Tumour and Microenvironment Interactions Group, Institute of Biomedical Engineering (INEB), Institute for Research and Innovation in Health (i3S), University of Porto, Rua Alfredo Allen 208, 4200-135 Porto, Portugal; patricia.cardoso@i3s.up.pt (A.P.C.); mariajo@ineb.up.pt (M.J.O.); 5 Institute of Biomedical Sciences Abel Salazar (ICBAS), Rua Jorge de Viterbo Ferreira 228, 4050-313 Porto, Portugal

**Keywords:** iNKT cells, macrophages, CD1d, CD40L, immunomodulation

## Abstract

CD1d-restricted invariant Natural Killer T (iNKT) cells are unconventional innate-like T cells whose functions highly depend on the interactions they establish with other immune cells. Although extensive studies have been reported on the communication between iNKT cells and macrophages in mice, less data is available regarding the relevance of this crosstalk in humans. Here, we dove into the human macrophage-iNKT cell axis by exploring how iNKT cells impact the survival and polarization of pro-inflammatory M1-like and anti-inflammatory M2-like monocyte-derived macrophages. By performing in vitro iNKT cell-macrophage co-cultures followed by flow cytometry analysis, we demonstrated that antigen-stimulated iNKT cells induce a generalized activated state on all macrophage subsets, leading to upregulation of CD40 and CD86 expression. CD40L blocking with a specific monoclonal antibody prior to co-cultures abrogated CD40 and CD86 upregulation, thus indicating that iNKT cells required CD40-CD40L co-stimulation to trigger macrophage activation. In addition, activated iNKT cells were cytotoxic towards macrophages in a CD1d-dependent manner, killing M1-like macrophages more efficiently than their naïve M0 or anti-inflammatory M2-like counterparts. Hence, this work highlighted the role of human iNKT cells as modulators of macrophage survival and phenotype, untangling key features of the human macrophage-iNKT cell axis and opening perspectives for future therapeutic modulation.

## 1. Introduction

Invariant Natural Killer T (iNKT) cells are unconventional innate-like T lymphocytes that contribute to regulate both homeostatic and/or pathogenic immune responses [1,2]. They are restricted to CD1d, a non-polymorphic major histocompatibility complex (MHC) class I-like molecule that is expressed by antigen-presenting cells (APCs) [3]. iNKT cells express a semi-invariant T cell receptor (TCR) that recognizes a broad range of endogenous and exogenous lipid antigens, being α-Galactosylceramide (α-GalCer; αGC) their prototype agonist [2]. Alternatively, another TCR-independent mechanism of iNKT activation embraces responses to interleukin (IL)-12 and/or IL-18 [4,5,6].

Upon activation, iNKT cells are characterized by a rapid and pronounced secretion of both Th1- and Th2-like cytokines, including interferon-gamma (IFN-γ), granulocyte macrophage-colony stimulating factor (GM-CSF), tumor necrosis factor (TNF)-α, IL-2, IL-4, IL-13 and IL-17 [2]. In fact, iNKT-derived inflammatory cytokines (e.g., IFN-γ and TNF-α) have been shown to trigger neutrophil recruitment and/or transactivation of CD8^+^ T cells, dendritic cells or macrophages in the context of *Pseudomonas aeruginosa* [7], *Streptococcus pneumoniae* [8,9], *Listeria monocytogenes* [10], *Chlamydia pneumoniae* [11,12], cytomegalovirus [13] and Influenza A virus [14,15] infections, as part of a protective response against infectious agents. In addition, others have also reported a crucial role for IL-4 released by iNKT cells in germinal centers formation and B cell immunity initiation during Influenza infection [5]. Aside from transactivating other immune cells, iNKT cells also exhibit cytotoxic properties and may therefore directly target and kill infected cells, including monocytes [16,17], macrophages [18] or dendritic cells [19] via the Fas-FasL pathway or through IFN-γ and cytotoxic granule release [6]. Due to these cytotoxic and immunomodulatory functions, iNKT cells have been further described as central participants of antitumor immunity, not only by directly targeting CD1d-expressing malignant cells for cytolysis [20,21,22,23,24,25], but also by killing or reprogramming pro-tumor immunosuppressive myeloid cells in the tumor microenvironment [26,27].

Importantly, iNKT cells ultimate functions in the tissues are largely shaped by the interactions they establish with other immune cells, including macrophages [28,29]. Macrophages are highly plastic phagocytes whose functional phenotype is critically modulated by the inflammatory milieu they are inserted in [30,31,32]. Macrophage activation stages vary along a full continuous spectrum of several inflammatory phenotypes, with the M1- and M2-like profiles representing either ends of this spectrum [31,33]. M1-like macrophages emerge in response to a pro-inflammatory environmental challenge and thus exhibit a marked secretion of pro-inflammatory cytokines, including type I IFN, IL-12, IL-1β and IL-6, and also Th1-attracting chemokines [30,31,32,34]. Hence, whereas they are generally characterized by crucial microbicidal and tumoricidal activities, they may also drive aberrant inflammation leading to tissue damage and autoimmune responses [30,34]. In contrast, in response to Th2-like anti-inflammatory cues, macrophages typically polarize into an M2-like phenotype, characterized by the strong secretion of anti-inflammatory cytokines, such as IL-10 and TGF-β, and upregulation of arginase and extracellular matrix-related proteins [30,31,32,34]. Due to this, they have been extensively implicated not only in tissue repair and wound healing, but also in tumor promotion and fibrosis [30,34].

Over the past years, accumulating evidence has been uncovering the relevance of the macrophage-iNKT cell crosstalk in several murine models of disease. Firstly, iNKT cells can be activated by macrophages via CD1d-dependent lipid antigen presentation. Macrophages have been described to activate iNKT cells under steady state conditions in the thymus [35], lymph nodes [36], spleen [37,38,39], liver [37,40] and intestine [41], but also during bacterial [42,43,44,45], parasitic [46] and viral [5,47] infections. At the same time, iNKT cells may simultaneously modulate macrophage phenotypic and functional signatures. Whereas iNKT cell activation was shown to trigger M2 polarization of adipose tissue macrophages in murine models of obesity [48,49], others have proposed M1-promoting and M2-suppressing roles for iNKT cells in metaflammation [50,51] or cancer [27,52,53,54]. Hence, depending on each cell subpopulation, the interaction site and the pathophysiological context, the outcomes of this interplay may be substantially diverse [28,29].

Although extensive studies have been reported on the murine macrophage-iNKT cell axis, less data is available regarding the relevance of this crosstalk in humans. Considering this background, this study aimed to dive into the human macrophage-iNKT cell crosstalk by investigating the effects of human iNKT cells on the survival and activation of distinct human macrophage subsets (M0, M1- and M2-like). Herein, we demonstrate that human iNKT cells induce macrophage activation with contributions from both CD1d and CD40L, while also mediating subset-dependent macrophage death via CD1d engagement.

## 2. Materials and Methods

### 2.1. Ethics Statement

Human buffy coats were kindly provided by the Immunohemotherapy Service of Centro Hospitalar Universitário São João (CHUSJ), Porto, Portugal, after Hospital Ethics Committee approval (reference 90/19) and individual signed consent, following strictly the recommendations of the European Union Directive 2010/63/EU and the Helsinki declaration.

### 2.2. Human Monocyte Isolation and Macrophage Differentiation and Polarization

Human CD14^+^ cells were isolated from buffy coats from healthy blood donors kindly provided by the Immunohemotherapy Service of CHUSJ by a negative selection approach, as previously described [55]. Briefly, buffy coats were centrifuged at 1200× *g* for 30 min at room temperature and without brake. The resulting cloudy interface containing peripheral blood mononuclear cells (PBMCs) was collected and incubated with RosetteSep Human Monocyte Enrichment Cocktail (StemCell Technologies, Vancouver, BC, Canada) for 20 min in a roller agitator at room temperature. This mixture was diluted 1:1 with phosphate-buffered saline (PBS) 2% fetal bovine serum (FBS) (Gibco, Grand Island, NY, USA), layered onto Histopaque-1077 (Sigma-Aldrich, St. Louis, MO, USA) in a 2:1 proportion and centrifuged at 1200× *g* for 30 min, without brake. The monocyte-enriched ring was then collected and washed three times with PBS, alternated with centrifugations of 5 min at 1300 rpm. Isolated cells were resuspended in RPMI 1640 (Gibco, Grand Island, NY, USA) 1% Pen/Strep (Gibco, Grand Island, NY, USA) supplemented with 5% human serum (HS) (Invitrogen, Grand Island, NY, USA) or 10% FBS and hM-CSF (50 ng/mL) (ImmunoTools, Friesoythe, Germany) and seeded in 6-well culture plates on top of a glass coverslip at a density of 3 × 10^6^ cells/well. At day 7 of culture, culture medium was replaced by fresh RPMI 1% Pen/Strep with 5% HS or 10% FBS and without hM-CSF to enable differentiated macrophages resting for 3 days. Ten days after isolation, macrophages were then polarized into the M1- or M2-like phenotypes through a 3-day LPS (15 ng/mL) (Sigma-Aldrich, St. Louis, MO, USA)/LPS (10 ng/mL) + IFN-γ (50 ng/mL) or IL-10 (15 ng/mL) (both from ImmunoTools, Friesoythe, Germany) stimulation, respectively, or left unstimulated to maintain the naïve M0 profile.

### 2.3. Human iNKT Cell Culture

The human iNKT cells used were expanded from a primary culture generated in our group as previously described [3]. iNKT cells were cultured in 24-well plates with RPMI 1640, Sodium Pyruvate, Kanamycin and Non-Essential Amino Acids (all from Gibco, Grand Island, NY, USA) supplemented with 5% of human AB serum and 100 U/mL of recombinant human IL-2 (kindly provided by NCI, USA). For expansion, iNKT cells were re-stimulated with irradiated PBMCs and 1 μg/mL of phytohemagglutinin (PHA) (Thermo Fischer Scientific, Grand Island, NY, USA) every 17–20 days when they stopped proliferating.

### 2.4. Macrophage-iNKT Cell Co-Culture Assays

Co-cultures were performed in 6-well plates following macrophage polarization for 18 h in a 1:4 iNKT cell/macrophage ratio and in the presence of 0, 4 or 60 of α-GalCer (KRN7000, Sigma, St. Louis, MO, USA). iNKT cells were used on days 14–17 post re-stimulation. For CD1d and CD40L (CD154) blocking experiments, macrophages were pre-incubated with a specific anti-CD1d monoclonal antibody (clone 51.1, 10 μg/mL) (BioLegend, San Diego, CA, USA) for 1 h. Likewise, iNKT cells were incubated with a specific anti-CD154 monoclonal antibody (clone 24–31, 20 μg/mL) (BioLegend, San Diego, CA, USA) for 1 h prior to co-culture. Then, co-cultures were carried out in the presence of CD1d or CD40L antibodies and α -GalCer (60 nM) antigen for 18 h. Upon co-culture, culture medium containing iNKT cells/detached macrophages was recovered and centrifuged at 670× *g* for 5 min to separate cells from the actual supernatants. For macrophage detachment, wells were washed with PBS and macrophages were incubated with accutase (GRiSP) for 35 min at 37 °C, 5% CO_2_. Each well was then gently scrapped and washed with cold PBS and detached macrophages were recovered to the corresponding tubes containing the pellet of iNKT cells. Cells were finally resuspended in PBS, 2% FBS, 1 mM EDTA, 0.1% NaN_3_ and kept at 4 °C until staining for flow cytometry analysis.

### 2.5. Flow Cytometry

For surface markers staining, recovered cells were firstly incubated with an FcR blocking reagent (Miltenyi, Cologne, Germany) for 10 min to prevent non-specific antibody binding to FcR receptors and then stained with fluorochrome-conjugated monoclonal antibodies (mAbs) diluted in PBS, 2% FBS, 1 mM EDTA, 0.1% NaN_3_ for 20 min, in the dark, at 4 °C. Unless specified, all antibodies were purchased from BioLegend, San Diego, CA, USA. The following mAbs were used: anti-human CD40 (clone 53C), anti-human CD86 (clone BU63), anti-human CD163 (clone GHI/61), anti-human CD14 (clone OFC14D, ImmunoTools, Friesoythe, Germany), anti-human CD3 (clone UCHT1, eBioscience, San Diego, CA, USA), anti-human CD4 (clone OK74), anti-human CD8 (clone RPA-T8, eBioscience, San Diego, CA, USA). PBS57-loaded CD1d tetramer was kindly provided by NIH core tetramer facility. Dead cells were excluded through Fixable Viability Dye (eBioscience, San Diego, CA, USA) staining. Cells were fixed in 1% paraformaldehyde (PFA) (Agar Scientific, Stansted, UK) for 15 min, acquired on BD FACSCantoII and analysed with FlowJo V10 (BD Biosciences, San Diego, CA, USA).

For intracellular staining, cells were fixed with 2% PFA for 10 min and permeabilized in PBS Saponin (PBS 2% FBS 1 mM EDTA 0.01% NaN_3_ 0.5% Saponin (Sigma, St. Louis, MO, USA)) for 5 min. Upon permeabilization, samples were stained with fluorochrome-conjugated mAbs diluted in PBS Saponin. The following mAbs were used: anti-human granzyme B (clone GB11), anti-human T-bet (clone 4B10, eBioscience, San Diego, CA, USA), anti-RORγt (clone AFKJS.9, eBioscience, San Diego, CA, USA). Cells were then fixed, acquired and analyzed as aforementioned for extracellular staining. For granzyme B detection, cells were incubated with 10 μg/mL of brefeldin A (BFA) (Sigma, St. Louis, MO, USA) for 6 h prior to staining and kept in buffers with BFA until the fixation step.

### 2.6. Immunocytochemistry

Media from monocultures and co-cultures were removed from each well and samples were washed with PBS. Cells were then fixed in 4% paraformaldehyde for 20 min and stored at 4 °C. To stain samples, coverslips were washed and quenched for 10 min with 50 mM NH_4_Cl. Cells were permeabilized by incubating them with 0.2% Triton for 5 min. After washing, coverslips were incubated with 5% BSA (Sigma-Aldrich, St. Louis, MO, USA) solution for 30 min and transferred to a previously marked paraffin sheet. For staining, each coverslip was first incubated with mouse anti-α-tubulin antibody (Sigma-Aldrich, St. Louis, MO, USA T9026) for 1 h, followed by incubation with the secondary antibody, goat anti-mouse Alexa 488 (Invitrogen–Molecular Probes, Eugene, Oregon, USA), for 45 min. Actin was stained with Phalloidin-568 (Invitrogen–Molecular Probes, Eugene, Oregon, USA) for 20 min at room temperature. Every incubation was followed by 3 times washing with PBS under agitation for 5 min. Coverslips were turned with cells facing down to a drop of Vectashield with DAPI (Vector Laboratories Inc., Newark, CA, USA) to counterstain the nuclei on identified glass slides. Coverslips were fixed using nail polish and stored at −20 °C in the dark. Visualization was performed at a Zeiss SP5 confocal microscope and images were analysed using ImageJ 1.43 software.

### 2.7. Enzyme-Linked Immunosorbent Assay (ELISA)

Levels of GM-CSF, IFN-γ, IL-4 and IL-10 on culture supernatants were assessed by ELISA. For IFN-γ, IL-4 and IL-10, ELISA kits from BioLegend San Diego, CA, USA were used according to the manufacturer’s instructions. GM-CSF concentration in the supernatants was measured using a purified anti-GM-CSF mAb (BVD2-23B6, BioLegend, San Diego, CA, USA) as capture antibody and a biotinylated anti-GM-CSF mAb (BVD2-21C11, BioLegend, San Diego, CA, USA) as detection antibody. Signal was detected by incubating plates with horseradish peroxidase-conjugated streptavidin (Invitrogen, Eugene, Oregon, USA), followed by o-phenylenediamine dihydrochloride (OPD) substrate (Sigma, St. Louis, MO, USA). Absorbances were read using a Bio-Tek (Winooski, Vermont, USA) uQuant microplate reader and the Gen5 software.

### 2.8. Correlation Analysis on the Cancer Genome Atlas Data

Correlation analysis was performed using GEPIA, a web server for analyzing the RNA sequencing expression data of 9736 tumors from The Cancer Genome Atlas (TCGA) project, using a standard processing pipeline [56]. Cancer type was set as colon adenocarcinoma. The similarity in gene expression patterns was evaluated and validated by the Correlation Analysis feature of GEPIA tool (assessed on the 28 June 2022). A pair-wise gene expression correlation analysis was performed for given sets of TCGA expression data, using the Pearson correlation coefficient.

### 2.9. Transmission Electron Microscopy

For transmission electron microscopy, 3 × 10^5^ CD14^+^ enriched cells were plated in an 8-well plate (Lab-Tek^®^ II CC2 TM) in 300 μL of medium. Macrophage differentiation, polarization and co-culture with iNKT cells were carried out as previously mentioned. Samples were fixed overnight with 2.5% glutaraldehyde/2% paraformaldehyde in cacodylate buffer 0.1 M (pH 7.4) and washed in 0.1 M sodium cacodylate buffer. Next, samples were fixed in 2% osmium tetroxide in 0.1 M sodium cacodylate buffer overnight, followed by a new overnight fixation in 1% uranyl acetate. Dehydration was performed in gradient series of ethanol solutions and propylene oxide and included in EPON resin by immersion of samples in increasing series of propylene oxide to EPON (till 0:1 ratio) for 60 min each. Sample inclusion in EPON resin was performed in a silicon mold. Sections with 60 nm thickness were prepared on a RMC Ultramicrotome (PowerTome) using a diamond knife and recovered to 200 mesh Formvar Ni-grids, followed by 2% uranyl acetate and saturated lead citrate solution. Visualization was performed at 80 kV in a JEOL JEM 1400 microscope (Japan) and digital images were acquired using a CCD digital camera Orious 1100 W (Tokyo, Japan).

### 2.10. Statistical Analysis

Statistical analysis was performed using GraphPad Prism 9 software. Variables were first tested for normality using the Shapiro–Wilk test. Statistically significant differences between groups were assessed by paired *t*-test, Wilcoxon test, one-way ANOVA or Brown–Forsythe and Welch ANOVA test, as annotated in each figure legend. One-sample *t*-test was used to identify normalized ratios significantly different than 1. Statistical significance was considered as follows: * *p* < 0.05; ** *p* < 0.01, *** *p* < 0.001; **** *p* < 0.0001.

## 3. Results

### 3.1. Activated Human iNKT Cells Induce Activation of the Distinct Human Macrophage Subpopulations

Firstly, we sought to investigate whether human iNKT cells are capable of modulating the activation and polarization status of distinct macrophage subpopulations. To this end, human monocyte-derived macrophages were polarized into the pro-inflammatory M1- or anti-inflammatory M2-like profiles through LPS/LPS+IFN-γ or IL-10 treatment, respectively, or left untreated to maintain the naïve M0 profile (Supplementary Scheme S1). Macrophage polarization status was confirmed by flow cytometry analysis according to expression of the M1 co-stimulatory markers CD86 and CD40 and of the M2 marker CD163. As expected, both LPS- and LPS+IFN-γ-stimulated macrophages to express higher levels of CD86 and of CD40 than their unstimulated and IL-10-treated counterparts, confirming the acquisition of an M1-like profile (Supplementary Figure S1). In contrast, macrophage expression of the M2 marker CD163 increased upon IL-10 stimulation, in comparison with that of LPS-treated macrophages, which was suggestive of an M2-oriented profile. Importantly, among the distinct macrophage populations, CD1d levels were similar (Supplementary Figure S1), except for LPS +IFN-γ-stimulated macrophages that express significantly higher levels of CD1d. After validating macrophages phenotypes upon in vitro polarization, we decided to explore the effect of iNKT cells on naïve, M1- or M2-like macrophages polarization. For that, macrophages were cultured with or without human granzyme B-expressing CD4^+^ iNKT cells (Supplementary Figure S2) for 18 h, in the presence or absence of the lipid antigen α-GalCer (60 nM), and macrophage status post co-culture was evaluated by flow cytometry (Supplementary Scheme S1). For this analysis, macrophages were firstly distinguished from iNKT cells based on size differences (Supplementary Figure S3), as iNKT cells are markedly smaller than macrophages. Importantly, in the absence of iNKT cells, α-GalCer by itself did not alter macrophage surface expression of CD86, CD40 and CD163 (Supplementary Figure S4A).

**Figure 1:**
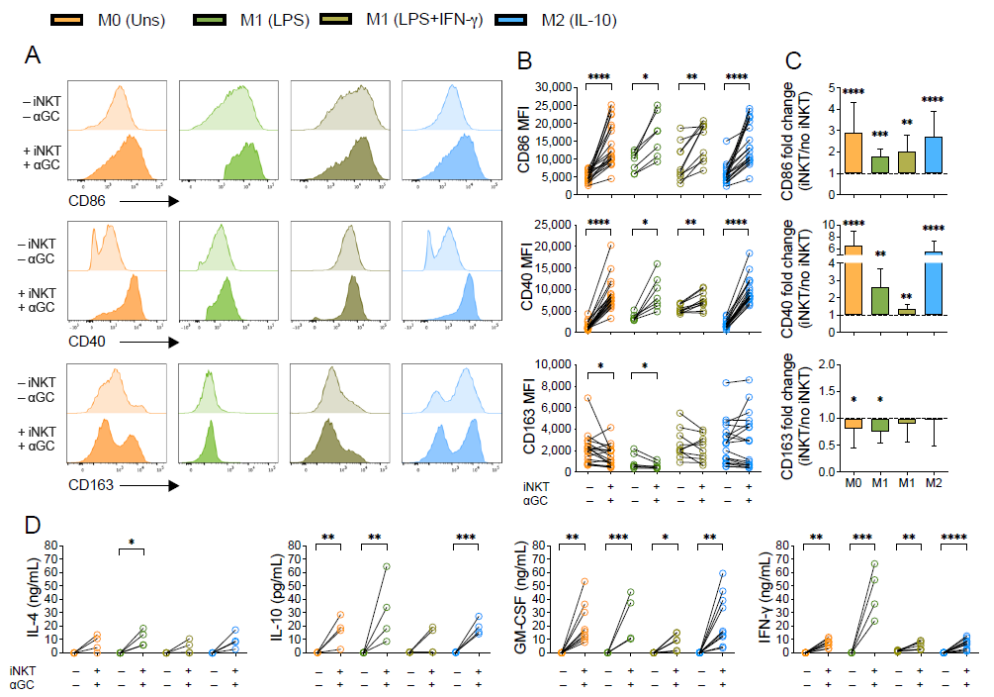
Human macrophages upregulate CD86 and CD40 expression upon co-culture with α-GalCer-activated iNKT cells. M0 (Unstimulated), M1- (LPS- or LPS+IFN-γ-stimulated) and M2-like (IL-10-stimulated) macrophages were co-cultured with or without iNKT cells plus α-GalCer (60 nM) for 18 h. (**A**) Representative flow cytometry plots of CD86, CD40 and CD163 expression on macrophages. (**B**) Mean fluorescence intensity (MFI) of CD86, CD40 and CD163 on macrophages with or without iNKT cells plus α-GalCer. (**C**) Relative macrophage CD86, CD40 and CD163 expression upon co-culture with α-GalCer-activated iNKT cells. Data was normalized to the respective monoculture condition (macrophages alone) for each subject. (**D**) IL-4, IL-10, GM-CSF and IFN-γ concentrations in supernatants from macrophage monocultures and co-cultures with iNKT cells plus α-GalCer. Cytokine levels were measured by ELISA. Data are representative of at least 4 independent experiments. * *p* < 0.05; ** *p* < 0.01; *** *p* < 0.001; **** *p* < 0.0001. (**B**) Wilcoxon test, (**C**) One-sample *t*-test (**D**) Paired *t*-test or Wilcoxon test.

Our data demonstrates that α-GalCer-activated iNKT cells triggered activation of M0, M1- and M2-like macrophage subpopulations, leading to upregulation of CD86 and CD40 expression (Figure 1A,B). The increase in CD86 expression upon co-culture with iNKT cells was more pronounced for M0 (fold change of 2.9 ± 1.4) and M2-(fold change of 2.7 ± 1.2) than for M1-like macrophages (fold change of 1.8 ± 0.3 for LPS; and 2.0 ± 0.8 for LPS+IFN-γ) (Figure 1C). Similar effects of iNKT cells were registered for CD40 expression, which increased around 6 times in M0 and M2-like macrophages when comparing macrophages cultured in the presence or absence of α-GalCer-activated iNKT cells, while the M1-like subsets exhibited a fold change of 2.6 ± 1.0 (LPS) and 1.3 ± 0.2 (LPS+IFN-γ). This is in accordance with the fact that M1-like macrophages, being pro-inflammatory, already exhibit increased baseline expression of both CD86 and CD40 surface receptors prior to co-culture, in comparison with the other subsets (Figure 1A and Supplementary Figure S1). Along with this, M0 and LPS-stimulated macrophages cultured with α-GalCer-activated iNKT cells suffered a fold decrease of 0.8 ± 0.3 and 0.7 ± 0.2, respectively, on CD163 expression, while no major differences were found for the other subsets (Figure 1C). Of note, co-culture with iNKT cells in the absence of α-GalCer also prompted increased CD86 and CD40 expression by M0, LPS-stimulated and M2 macrophages and decreased CD163 expression by M0 macrophages, although these variations were more subtle (Supplementary Figure S4B), when comparing with the ones observed for α-GalCer-activated iNKT cells (Figure 1B). Hence, this data indicates that human iNKT cells induce macrophage activation regardless of their initial phenotype, shaping naïve M0 and anti-inflammatory M2-like macrophages towards a pro-inflammatory M1-skewed profile.

To further explore the generalized activation state in macrophage-iNKT cell co-cultures, we measured, through ELISA, the concentrations of different cytokines (GM-CSF, IL-4, IFN-γ and IL-10) in supernatants of macrophages cultured alone or in the presence of activated iNKT cells. Both IFN-γ and GM-CSF levels increased in supernatants from co-cultures performed in the presence of α-GalCer, regardless of the macrophage subset considered (Figure 1D). Interestingly, the highest increase on IFN-γ was observed in supernatants of M1 (LPS) macrophages in co-culture with iNKT stimulated with α-GalCer. In addition, IL-4 and IL-10 concentrations were significantly higher in supernatants from iNKT cell co-cultures with α-GalCer and LPS-treated macrophages (for IL-4) or unstimulated, LPS- and IL-10-stimulated macrophages (for IL-10). An equivalent non-significant tendency was also observed for the LPS-IFN-γ subset (Figure 1D). Although both IL-10 and IL-4 were detected in the supernatants, the absolute cytokine levels should be considered for the final cytokine balance per condition. Indeed, GM-CSF levels were remarkably high for all macrophage subsets. Whereas M0 and M2-like macrophages exhibit comparable levels of IL-4, IL-10 and IFN-γ, the concentrations of IFN-γ are extremely high in the LPS-stimulated M1 population, supporting the existence of a more pro-inflammatory environment. 

Aiming to explore the clinical significance of iNKT cells effects on macrophage polarization, we analyzed The Cancer Genome Atlas (TCGA) expression data to determine the correlation between iNKT cells gene signature (TRAV10 and TRBV25-1, coding for iNKT prototypical TCR α- and β-chains) and both M1- (CD40, HLA-DRA, CD80 and CD86) and M2-like macrophages (CD206 and CD163) gene signatures, in a cohort of colon adenocarcinoma and paired adjacent normal tissue. Colon cancer was chosen for this analysis given that iNKT cells infiltration in the tumor bed has been reported as a favorable prognostic factor [57], whereas infiltration of macrophages, especially those with an immunosuppressive phenotype, was mainly associated with advanced colorectal cancer stages [58,59,60]. As a control for total T lymphocytes, correlation between CD3 expression and both M1 and M2-like macrophage signatures was evaluated.

As shown in Table 1, both TRAV10 and TRBV25-1 mRNA expression levels were positively correlated with the M1 signature in both tumor (R = 0.49 and R = 0.67, respectively) and normal adjacent tissue (R = 0.52 and R = 0.49, respectively), while in the case of the M2 signature this moderate positive correlation was only found in the tumor tissue (R = 0.40 and R = 0.54, respectively). Importantly, the strongest correlation coefficient was found when comparing gene expression patterns of TRAV10 and TRBV25-1 genes together (iNKT cell signature) with the M1 signature transcripts in the tumor tissue (R = 0.79), which was higher than total T cells (CD3) and M1 signature (R = 0.68), emphasizing the relevance of iNKT cells and M1-like macrophages proximity in cancer. This correlation is weaker when looking at the TRAV10 and TRBV25-1 genes together with the M2 signature genes in the tumor site (R = 0.63) and inexistent in the normal adjacent tissue (R = 0.064). Therefore, iNKT cells expression patterns seem to correlate better with those of M1- other than with M2-like macrophages in both normal and tumoral tissue.

### 3.2. Activation of Macrophages by iNKT Cells Is Modulated by CD40L

**Figure 2:**
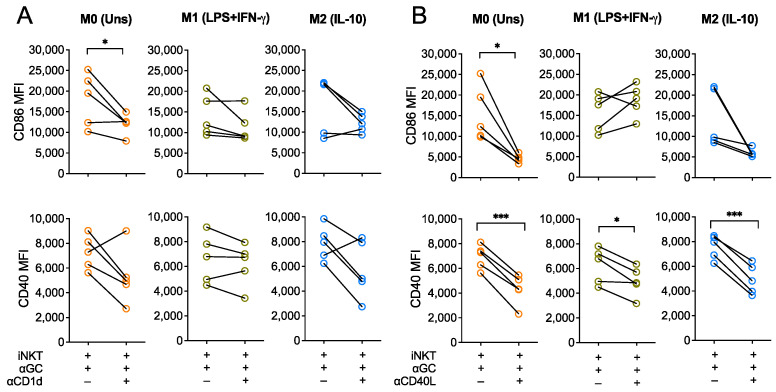
CD40L engagement is required for iNKT-mediated activation of macrophages. CD86 and CD40 expression (MFI) of M0 (Unstimulated), M1- (LPS+IFN-γ-stimulated) and M2-like (IL-10-stimulated) macrophages were co-cultured with iNKT cells and α-GalCer (60 nM) for 18 h in the presence or absence of (**A**) anti-CD1d or (**B**) anti-CD40L blocking antibodies, added to the medium 1 h prior co-cultures. Data are representative of 5 independent experiments. * *p* < 0.05; *** *p* < 0.001. Paired *t*-test.

Since α-GalCer is presented to iNKT cells by CD1d, we investigated whether TCR engagement was required for the iNKT-driven activation of macrophages by blocking CD1d during co-cultures. To do so, polarized macrophages were pre-incubated with an anti-CD1d monoclonal antibody (clone 51.1, 10 μg/mL) for 1 h. Then, co-cultures were carried out for 18 h in the presence of α-GalCer (60 nM) and CD1d blocking antibody. During these blocking assays, M0, M1- and M2-like macrophages markedly increased CD86 and CD40 expression upon co-culture with iNKT cells, as we had previously observed (Figure 1). CD1d blockade seemed to partially abrogate this effect, since it resulted in decreased expression of both activation markers expression by the three macrophage subpopulations in 4 out of the 5 donors tested, although this tendency only reached statistical significance for M0 macrophage CD86 expression (*p* < 0.05) (Figure 2A). This suggests that CD1d engagement seems to be required for iNKT cells to induce macrophage activation upon co-culture.

In addition, considering that CD40 is important for iNKT cells co-stimulation and activation and that it was markedly upregulated in macrophages upon interaction with iNKT cells, we hypothesized that the CD40-CD40L co-stimulatory axis played important positive feedback on overall iNKT-induced macrophage activation. To address this, iNKT cells were firstly incubated with an anti-CD40L antibody (clone 24–31, 20 μg/mL) for 1 h and co-cultures were then carried out as previously explained in the presence of the CD40L blocking antibody. CD86 expression by M0 macrophages upon CD40L blocking was significantly lower (*p* < 0.05), when comparing with the co-culture condition without blocking (Figure 2B). In addition, preventing CD40-CD40L interactions between both cells also blocked CD40 upregulation by M0, M1- and M2-like macrophages (Figure 2B). Altogether, this suggests that iNKT-mediated activation of macrophages is mediated by CD1d and, to a higher extent, by CD40, thus underpinning key roles of CD1d-restricted antigen presentation and CD40-CD40L co-stimulation on iNKT cells capacity to shape macrophage activation status and subsequent phenotype.

### 3.3. Activated iNKT Cells Kill more Efficiently M1-like Than M2-like Macrophages

**Figure 3:**
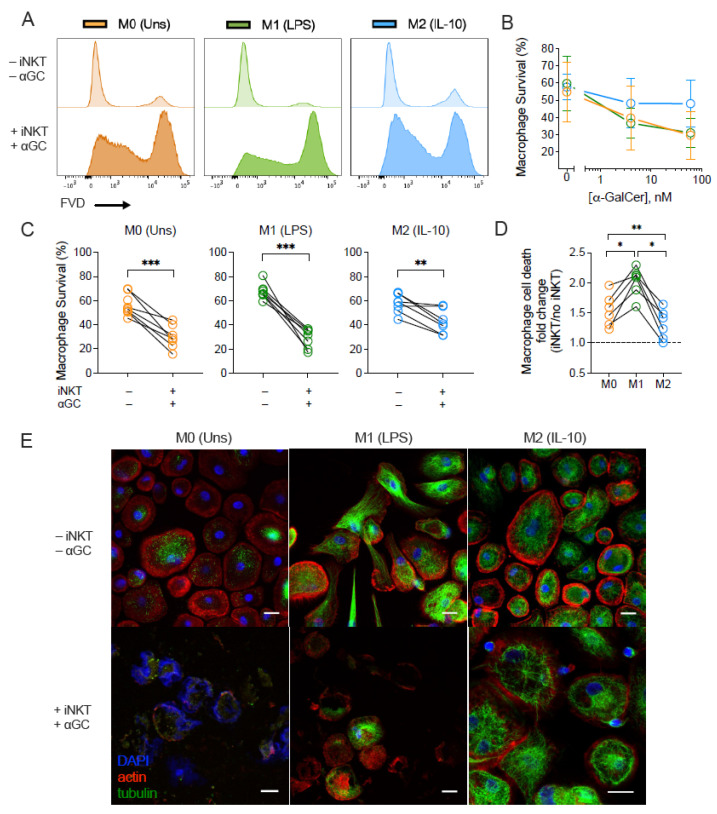
iNKT cells induce subset-dependent cell death of macrophages. M0 (Unstimulated), M1- (LPS-stimulated) and M2-like (IL-10-stimulated) macrophages were co-cultured with or without iNKT cells plus α-GalCer for 18 h. (**A**) Representative flow cytometry plots of macrophage viability. (**B**) Survival of M0, M1- and M2-like macrophage subsets in the presence of iNKT cells activated with 0, 4 or 60 nM of α-GalCer. (**C**) Survival of M0, M1- and M2-like macrophages in the presence of iNKT cells activated with 60 nM of α-GalCer. (**D**) Fold change of α-GalCer-driven iNKT cells killing capacity against macrophages. Macrophage cell death upon co-culture was normalized to the respective monoculture condition for each subject. (**E**) Immunocytochemistry of M0, M1- and M2-like macrophages after 18 h incubation with activated iNKT cells. Representative pictures of 1 subject, taken with a 400× magnification, using a Leica confocal SP5 microscope. Scale bar: 20 μm. Data are representative of (**B**) 3, (**C**,**D**) 7 independent experiments. * *p* < 0.05; ** *p* < 0.01; *** *p* < 0.001. (**B**) Two-way ANOVA, (**C**) Paired *t*-test, (**D**) One-way ANOVA, Tukey’s multiple comparisons test.

To address the killing capacity of iNKT cells over distinctly polarized macrophages, we incubated M0, M1- and M2-like macrophage subsets in the presence or absence of iNKT cells and α-GalCer (4 and 60 nM). Macrophage survival was measured by flow cytometry using a fixable viability dye (Figure 3 and Supplementary Figure S3). Incubating α-GalCer or iNKT cells alone did not influence the viability of the tested macrophage subsets (Supplementary Figure S5). However, following co-culture with iNKT cells and increasing concentrations of α-GalCer (4 and 60 nM), macrophage viability was reduced (Figure 3B). Antigen titration suggested that, at 60 nM of α-GalCer, the M2-like population survived better than M0 and M1-like subsets, but this effect was not as remarkable in the condition with 4 nM of antigen. Therefore, further experiments were performed using 60 nM of α-GalCer. Addition of iNKT cells plus α-GalCer led to a reduction in the survival of all macrophage subsets (Figure 3C), although the extension of iNKT cell killing capacity varied in the presence of the various subsets. Indeed, α GalCer-driven iNKT cells induced distinct rates of macrophage cell death, which diverged according with the macrophage profile. When compared with the baseline survival of macrophages when cultured alone, iNKT cells induced in average 2.02 ± 0.09 times more killing of M1-like population. Instead, their impact on M0 and M2-like macrophages was significantly lower (1.54 ± 0.11 and 1.31 ± 0.10, respectively) (Figure 3D). The differential effect of activated iNKT cells on M0, M1- and M2-like populations was also confirmed by immunocytochemistry. After 18 h of co-culture, activated iNKT cells induced cell death of both M0 and M1-like macrophages, leading these populations to lose cell integrity (Figure 3E). Macrophage actin and tubulin structures were found to be disrupted in the presence of activated iNKT with no traces of nuclear material (DAPI^−^ staining), in the case of M1-like, or with the nuclear material dispersed in the cytoplasmatic space, as observed in the case of M0 subset, whereas the M2-like subset kept cell integrity (Figure 3E).

### 3.4. Macrophage Killing by Activated iNKT Cells Requires CD1d

**Figure 4:**
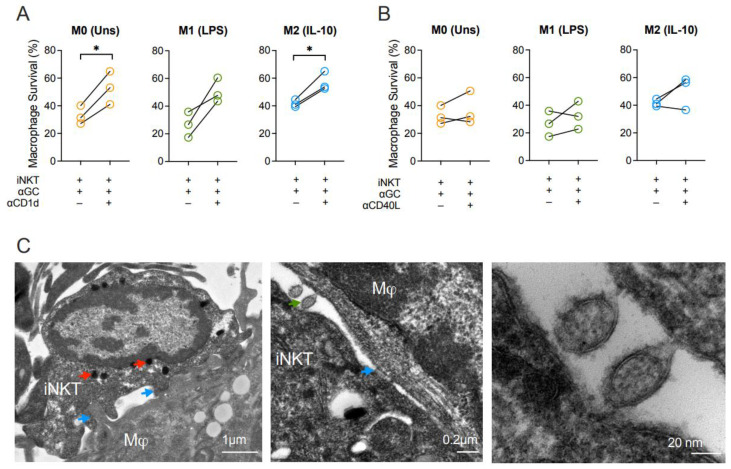
Cytotoxicity of iNKT cells towards macrophages is mediated by CD1d. Survival of M0 (Unstimulated), M1- (LPS-stimulated) and M2-like (IL-10-stimulated) macrophages co-cultured with iNKT cells and α-GalCer (60 nM) for 18 h in the presence or absence of (**A**) anti-CD1d or (**B**) anti-CD40L blocking antibodies. (**C**) Transmission electron microscopy of synapse between an iNKT cell and a macrophage after 18 h of co-culture. Blue arrow: synapse, red arrow: charged vesicles, green arrow: vesicular exchange. Representative pictures of 1 subject, taken with a 15,000× (left), 40,000× (middle) and 300,000× (right) magnification, using a CCD digital camera Orious 1100 W. Scale bar: 1 μm (left), 0.2 μm (middle), 20 nm (right). Data are representative of (**A**,**B**) 3 independent experiments. * *p* < 0.05. Paired *t*-test.

To unveil the mechanisms underlying macrophage killing by iNKT cells, we firstly blocked CD1d. Blocking of CD1d increased the survival rates of M0 and M2-like macrophages in the presence of α-GalCer-driven iNKT cells (Figure 4A). Although survival of the M1-like subset also tended to be recovered, this effect was not statistically significant (Figure 4A). Additionally, considering the upregulation of CD40 on all macrophage subpopulations and its influence on macrophage activation, we decided to evaluate the roles of CD40 on macrophage survival. CD40L blocking consistently showed that CD40-CD40L interactions did not directly influence macrophage death induction (Figure 4B). These results evidence that iNKT cells’ ability to induce macrophage death requires CD1d but occurs in a CD40L-independent mechanism. By transmission electron microscopy, we were able to visualize in detail the immunological synapse (Figure 4C) where a macrophage and an iNKT cell interact with each other through direct cell membrane contact (blue arrows). Of note, the iNKT cell contains highly dense vesicles, likely carrying granzymes and other molecules (red arrows) with effector functions. Moreover, it is possible to observe the vesicle exchange (green arrow) between the two cell types, that might be important for the outcome of this interaction.

## 4. Discussion

The importance of the macrophage-iNKT cell crosstalk has been stated under several pathophysiological contexts, playing protective or deleterious roles depending on the macrophage status and disease models considered [29]. Cortesi et al. described that murine iNKT cells favor the M1-like population while killing M2-like macrophages, an observation that can be extremely relevant to bypass the anti-inflammatory environment in the tumor site [27]. Nevertheless, studies so far have not approached this interaction using human cells. Therefore, this work provides important insights that point out the capacity of α-GalCer-driven human iNKT cells to: (1) activate all M0, M1- and M2-like macrophages; and (2) kill more efficiently M1-like in comparison with the M2-like subset, employing CD1d- and CD40-mediated mechanisms.

Overall, the three human macrophage subsets (M0, M1- and M2-like) exhibited upregulation of CD40 and CD86 co-stimulatory receptors upon co-culture with activated iNKT cells, although at different extensions. The relative increase in the expression of these pro-inflammatory markers was more marked for M0 and M2-like populations, since their baseline levels are lower than those of both M1-like subsets. It is important to note that in the absence of α-GalCer, iNKT cells induced upregulation of CD86 and CD40 in M0, LPS-stimulated M1- and M2-like macrophages, suggesting either the possibility of endogenous antigen recognition from iNKT cells or TCR-independent mechanisms of stimulation. Importantly, the generalized activation of macrophages by iNKT cells was shown to be mediated by CD40-CD40L co-stimulation, since blocking of CD40L attenuated macrophage CD40 and CD86 upregulation after co-culture with activated iNKT cells. Surprisingly, CD1d blocking did not significantly reduce surface expression of these activation markers on the different macrophage subsets, despite it tending to be dimer. This result might be related to an insufficient blocking of CD1d molecules, or due to other pathways of iNKT cell activation. Considering that significantly increased levels of GM-CSF, IFN-γ, IL-4 and IL-10 were found in the supernatants from co-cultures with α-GalCer, it is also plausible that, upon α-GalCer-driven activation, iNKT-derived cytokines also contribute to the modulation of macrophage phenotypes, apart from CD40-mediated co-stimulation. This is in accordance with previous reports showing that IL-10 [61], IL-4 [48,49,62,63,64] or IFN-γ [10,65] secreted by murine iNKT cells influence macrophage polarization and their corresponding effector functions. Overall, and similarly to previous in vitro reports with mouse-derived cells [27,63], this data demonstrates that activated human iNKT cells induce polarization of all macrophage subsets into a pro-inflammatory M1-skewed profile.

In addition, regarding the killing assays, there were also significant differences in macrophage survival in the presence of activated iNKT cells. Among the tested macrophage populations, the LPS-polarized subset was more efficiently killed by iNKT cells than M0 or M2-like subsets. Here, CD1d blocking was demonstrated to partially retrieve the diminishment of macrophage survival induced by iNKT cells for M0 and M2-like subsets. M1-like macrophages also tended to recover their viability in the presence of CD1d blocking antibodies, although not being significant. CD1d expression levels were similar for the three tested macrophage subsets, thus underlining that TCR-dependent mechanisms do not exclusively explain the differential macrophage killing. Even so, while anti-inflammatory M2-like macrophages seemed to be more protected from iNKT cells cytotoxicity, the M1-like subset endorsed a phenotypic and functional profile that rendered them more prone to iNKT cells killing. This M1-specific susceptibility might be explained by the fact that iNKT cells may be activated through other TCR-independent mechanisms. iNKT cells have been shown to be stimulated by IL-12 and/or IL-18 in mice with CD40 playing a role in the induction of pro-inflammatory cytokines such as TNF, IL-6, IFN-γ and IL-12p40 [4,26,66]. Since M1-like macrophages have higher baseline expression levels of the co-stimulatory molecules CD40 and CD86, and secrete more IL-6 and IL-12 than M0 or M2-like macrophages, this might comprise a network of mechanisms that enhance iNKT cell activation, tuning their proficient killer capacity. This alternative cytokine-mediated mechanism of activation could explain the insufficient blocking of macrophage activation by anti-CD1d antibodies, and ultimately, the incapacity to fully restore macrophage survival for all subsets. In addition, since the highest release of IFN-γ into the co-culture supernatants occurs when iNKT cells are co-cultured with LPS-induced macrophages, IFN-γ enrichment in these conditions may further act as an adjuvant to support iNKT effector cytotoxic functions. It is also possible that the partial blocking results from insufficient amount of antibody to totally block both macrophage activation and killing. Furthermore, although blockade of the CD40-CD40L axis impaired CD40 and CD86 expression on macrophage surface, thus buffering the activated state, this was not enough to abrogate iNKT cells cytotoxicity.

It still remains essential to dissect the mechanisms adopted by iNKT cells to exert their cytotoxic effect against differently polarized macrophages. Taking into consideration the transmission electron microscopy showing iNKT cells and macrophages communication through extracellular vesicles and the fact that these iNKT cells express granzyme B, it is feasible that this effect is dependent on the release of cytotoxic granules containing perforin and granzyme B. However, the roles of other potential pathways employed by iNKT cells involving FasL [27,67], TRAIL [68] and LIGHT/TNFS14 [69] should be addressed in the near future. Altogether, these results diverge from early observations in mice, in which iNKT cells protected M1-like macrophages and killed the M2-like ones [27]. However, there are discrepancies regarding cell origin and polarizing stimuli when comparing both studies. While Cortesi et al. used murine bone marrow-derived macrophages that were polarized into M1-like and M2-like subset using IFN-γ and IL-4, respectively [27], herein we used monocyte-derived macrophages from human blood polarized with LPS/LPS+IFN-γ to obtain the M1-like subset or with IL-10 to obtain M2-like subset. Moreover, Cortesi et al. polarized macrophages at higher concentrations for a shorter period of time (4 h) [27], contrasting with the long-lasting polarization (3 days) with lower doses of stimuli herein reported. In addition to possible distinct responses amongst species, these differences might lead to distinct phenotypes and functional capacities of each subset, further explaining the different outcomes.

Overall, this work highlights that iNKT cells skew M0 and M2-like macrophages into an M1-like profile, comprising a network of positive feedback loops that lead to a generalized activation state of macrophages. This capacity to push naïve M0 and anti-inflammatory M2-like macrophages into a pro-inflammatory profile may be extremely useful in pathological conditions that take advantage of an anti-inflammatory immune suppressive microenvironment to progress, such as cancer. Moreover, our data also suggests that, once a threshold of iNKT cell activation is reached, iNKT cells kill more efficiently pro-inflammatory macrophages, thus contributing to buffer a scenario of exacerbated inflammation. Moreover, considering that the bioinformatical analysis on TCGA data pointed to a strong correlation between iNKT cells and the M1 signature in the context of colon adenocarcinoma, it is crucial to further evaluate in in vivo models the interaction of these two immune subsets and scrutinize this axis in the tumoral context. Altogether, these results unravel key features of the human macrophage-iNKT cell axis whose manipulation may be relevant in disease, thus opening perspectives for future therapeutic modulation.

## Figures and Tables

**Table 1:** The Cancer Genome Atlas Program expression data analysis for the correlation between iNKT cells signature and M1- or M2-like macrophages signatures on colon adenocarcinoma and normal adjacent tissue.

	Colon Adenocarcinoma				Normal Adjacent Tissue			
	M1 Signature		M2 Signature		M1 Signature		M2 Signature	
	R	*p*	R	*p*	R	*p*	R	*p*
***CD3***	0.68	0	0.56	0	0.49	0.0012	0.022	0.89
***TRAV10***	0.49	0	0.40	2.9 × 10^−12^	0.52	5.0 × 10^−4^	0.23	0.16
***TRBV25-1***	0.67	0	0.54	0	0.49	0.0012	−0.0069	0.97
***TRAV10+TRBV25-1***	0.79	0	0.63	0	0.57	0.00011	0.064	0.69

M1-like macrophage signature—CD40, HLA-DRA, CD80 and CD86; M2-like macrophage signature—CD163 and CD206; iNKT cell signature—TRAV10 and TRBV25-1.

## Data Availability

Data supporting the findings of this study are available within the article and its Supplementary Materials. Raw data that are not already in the Supplementary Materials can be obtained from the corresponding author (M.F.M.) upon reasonable request.

## Supplementary Materials

The following supporting information can be downloaded at: https://www.mdpi.com/article/10.3390/biomedicines10071723/s1, Supplementary Scheme S1: Overview of the macrophage-Inkt cell co-culture assays. Supplementary Figure S1: Phenotypic characterization of human monocyte-derived macrophages upon polarization with LPS, LPS+IFN-γ or IL-10. Supplementary Figure S2: The human iNKT cells used for macrophage-iNKT cell co-culture experiments are CD4^+^CD8^−^GzmB^+^. Supplementary Figure S3: Gating strategy for flow cytometry analysis of the macrophage-iNKT cell co-culture assays. Supplementary Figure S4: iNKT cells induce macrophage upregulation of CD86 and CD40 in the absence of α-GalCer, while α-GalCer does not alter expression of CD86, CD40 and CD163 on all macrophage subpopulations. Supplementary Figure S5: Neither α-GalCer nor iNKT cells alter macrophage survival.
