# Supplementary material for: Human iNKT Cells Modulate Macrophage Survival and Phenotype
Source: Biomedicines. 2022 Jul 17;10(7):1723. doi: 10.3390/biomedicines10071723 (PMC9313099; doi:10.3390/biomedicines10071723)
Supplement: Supplementary file 1 [file biomedicines-10-01723-s001.zip › biomedicines-1770192-SI.pdf]

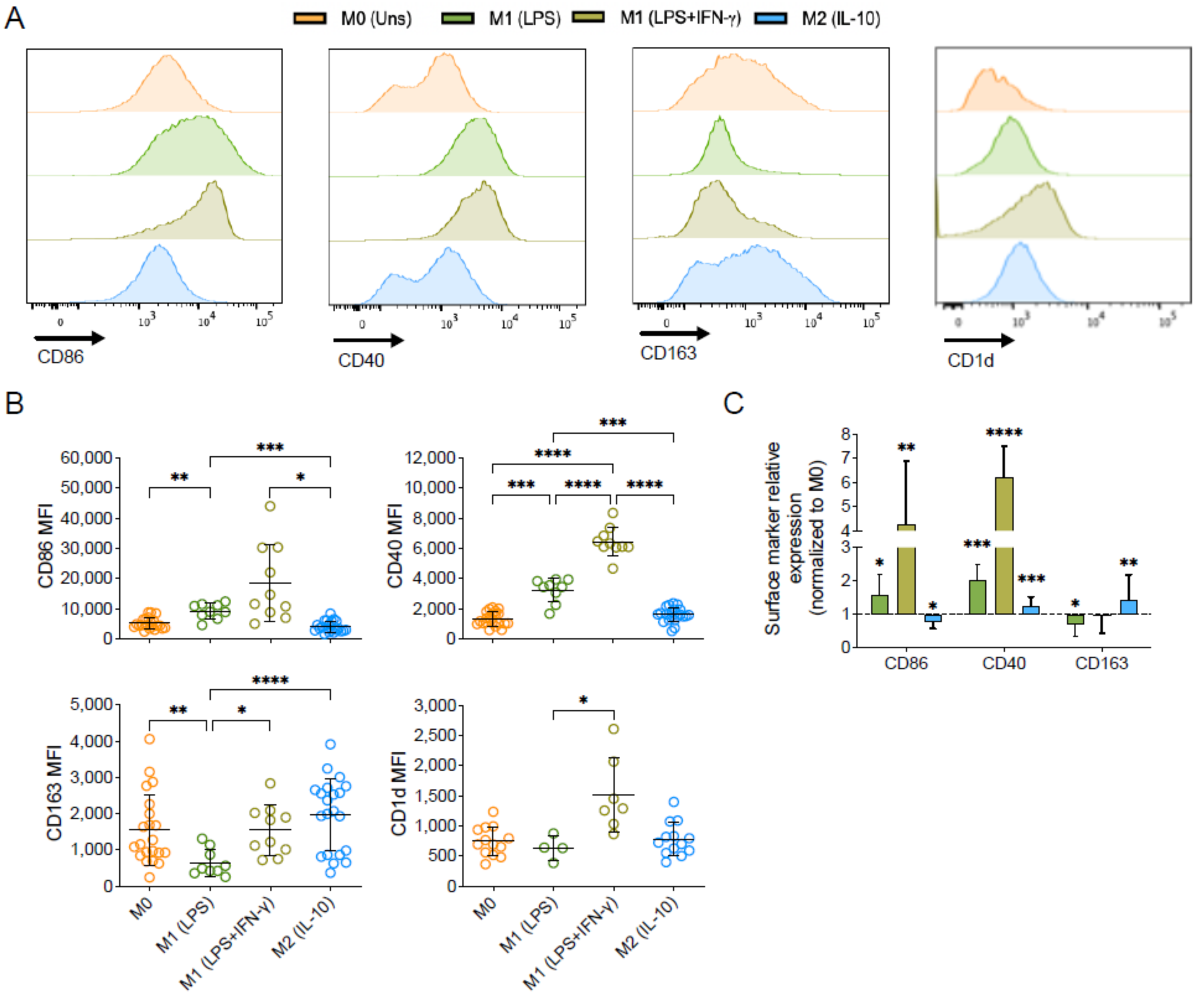

Figure S1. Phenotypic characterization of human monocyte-derived macrophages upon polarization with LPS, LPS+IFN-γ or IL-10. **(A)** Representative flow cytometry histograms for macrophage expression of CD86, CD40, CD163, CD1d surface molecules **(B)** CD86, CD40, CD163 and CD1d MFI of M0 (Unstimulated), M1- (LPS, LPS+IFN-γ) and M2-like (IL-10) macrophages. **(C)** Relative expression of CD40, CD86 and CD163 of M0 (Unstimulated), M1- (LPS, LPS+IFN-γ) and M2-like (IL-10) macrophages. MFI data was normalized to respective values of M0 (Unstimulated) macrophages. Data are represented as individual values, mean values and standard deviations of at least 9 independent subjects. *$p < 0.05$; **$p < 0.01$; ***$p < 0.001$; ****$p < 0.0001$. **(B)** Brown-Forsythe and Welch ANOVA test, Dunnet's T3 multiple comparison correction, **(C)** one-sample t test.

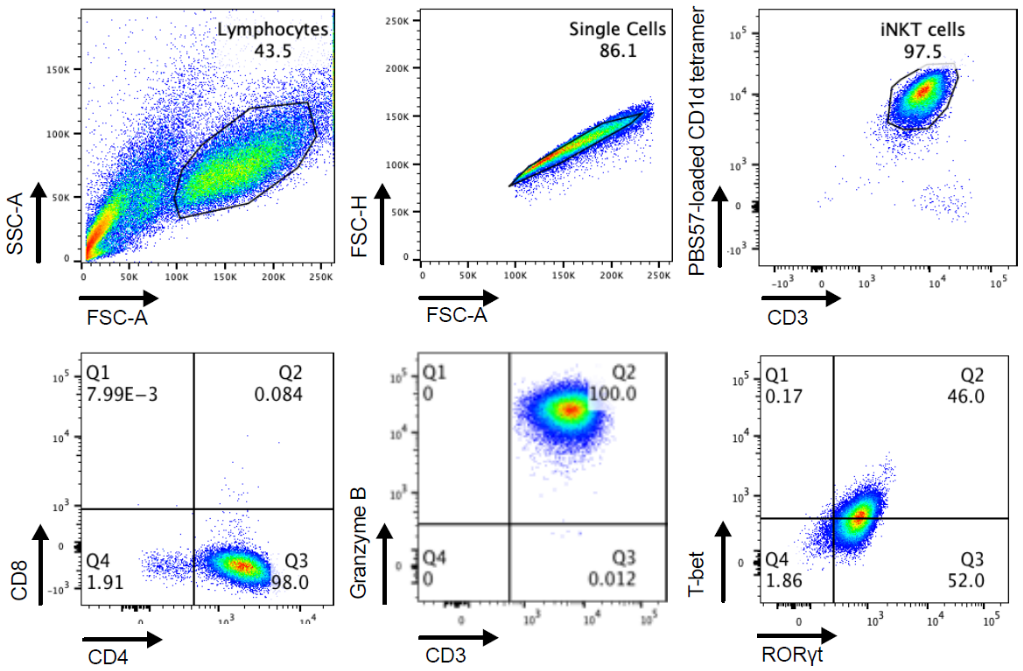

Figure S2. The human iNKT cells used for macrophage-iNKT cell co-culture experiments are CD4$^+$CD8$^-$ GzmB$^+$. Lymphocytes were gated based on size and granularity (FSC/SSC plot), followed by single cells selection (FSC-A/FSC-H plot). iNKT cells were gated on CD3$^+$, PBS57-loaded CD1d tetramer$^+$ cells (PBS57: α-GalCer analogue) and their phenotype was then assessed for CD4, CD8, Granzyme B, T-bet and RORγt expression.

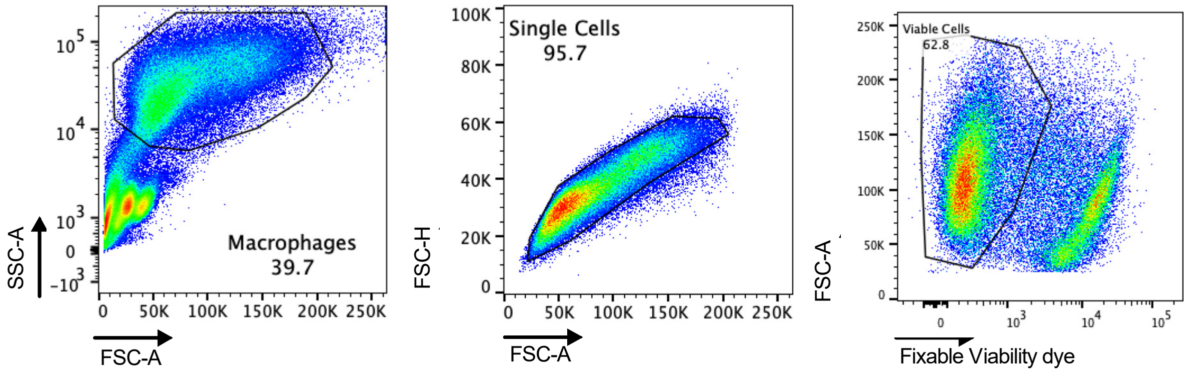

Figure S3. Gating strategy for flow cytometry analysis of the macrophage-iNKT cell co-culture assays. Gating strategy for flow cytometry analysis of macrophage viability and phenotype upon co-culture with iNKT cells. Macrophages were gated based on size (FSC-A/SSC-A plot), followed by single cell selection (FSC-A/FSC-H plot). Viable macrophages exhibiting low Fluorescence Viability Dye (FVD) fluorescence were gated and expression of each surface marker was analysed in the living cells.

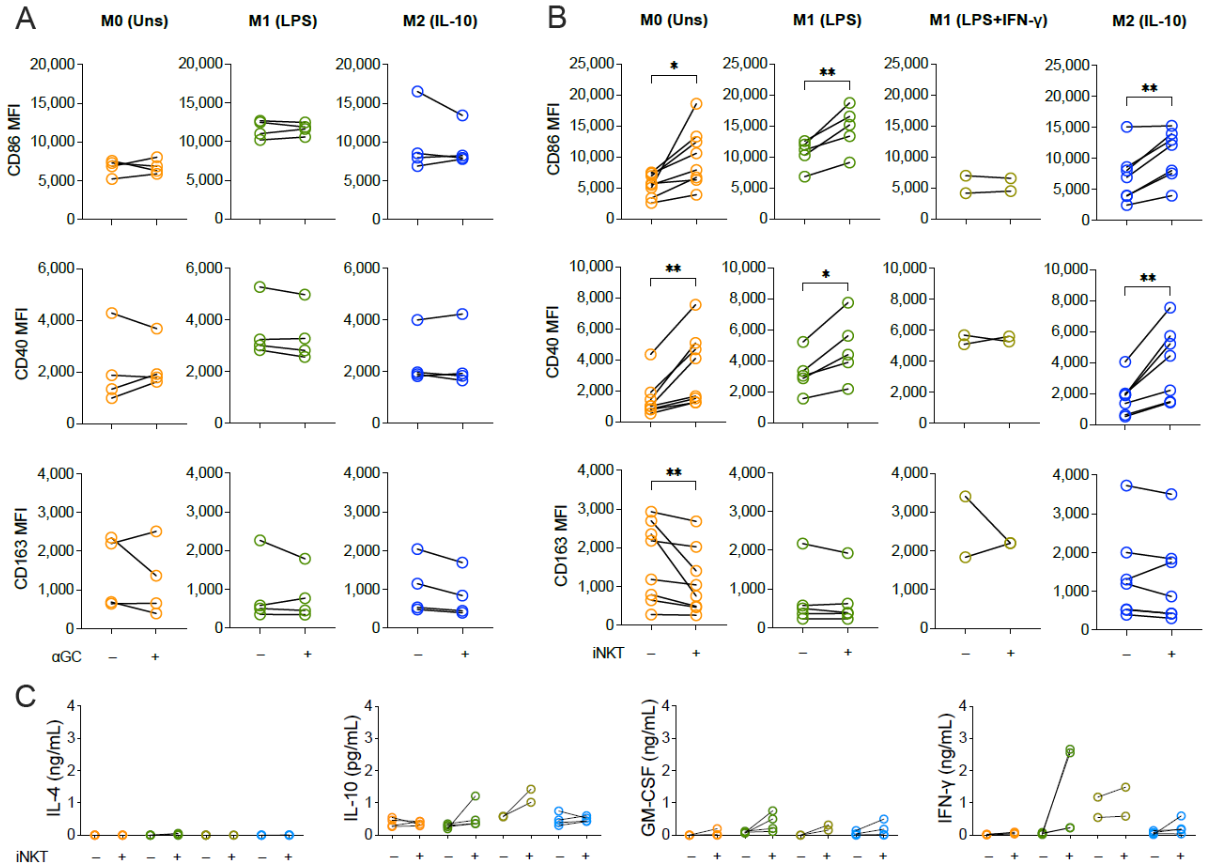

Figure S4. iNKT cells induce macrophage upregulation of CD86 and CD40 in the absence of α-GalCer, while α-GalCer does not alter expression of CD86, CD40 and CD163 on all macrophage subpopulations. CD86, CD40 and CD163 Mean Fluorescence Intensity (MFI) of M0, M1- and M2-like macrophages co-cultured with **(A)** α-GalCer (60 nM) or **(B)** iNKT cells for 18 hours. **(C)** IL-4, IL-10, GM-CSF, IFN-γ concentrations in supernatants from macrophage monocultures and co-cultures with iNKT cells without α-GalCer**.** Cytokine levels were measured by ELISA. $^{*}p < 0.05$; $^{**}p < 0.01$. Data are representative of **(A,C)** 4 or **(B)** at least 2 independent experiments. **(A,B)** Paired t-test, **(C)** Paired t-test or Wilcoxon test.

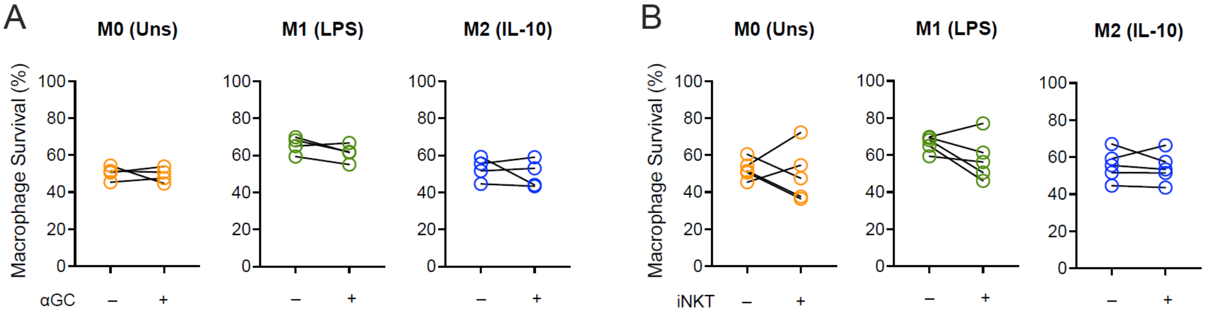

Figure S5. Neither α-GalCer nor iNKT cells alter macrophage survival. Survival of M0, M1- and M2-like macrophages incubated with **(A)** α-GalCer (60 nM) or **(B)** with iNKT cells for 18 hours. Data are representative of 4 independent experiments. Paired t-test.

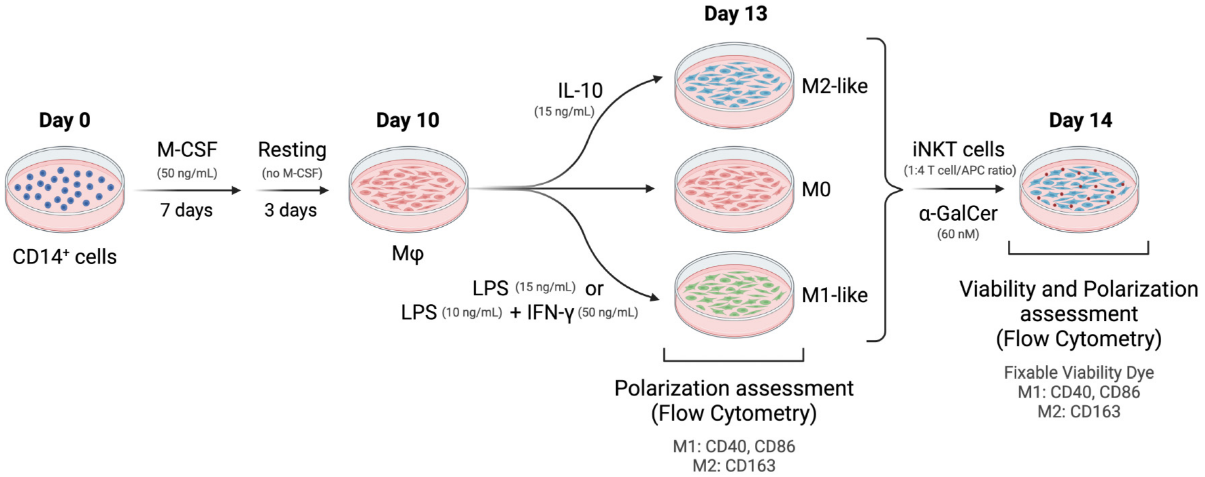

Scheme S1: Overview of the macrophage-iNKT cell co-culture assays. Schematic representation of the experimental setup for in vitro macrophage differentiation, polarization and subsequent co-culture with iNKT cells. Briefly, human CD14+ cells were isolated from buffy coats and differentiated into macrophages for 7 days in the presence of 50 ng/mL of M-CSF, followed by 3 days of resting in culture medium without M-CSF. Human monocyte-derived macrophages were then polarized into the M1-like profile by stimulation with LPS (15 ng/mL) or LPS (10 ng/mL) + IFN-γ (50 ng/mL); and into the M2-like phenotype through stimulation with IL-10 (15 ng/mL). No stimuli were added to maintain the naïve M0 macrophage profile. After 3 days, differently polarized macrophages were then cultured with or without human iNKT cells in a 1:4 T cell/macrophage ratio in the absence or presence of α-GalCer (60 nM). Upon 18 hours of co-culture, flow cytometry analysis was carried out to assess macrophages viability (based on Fixable Viability Dye staining) and phenotype (CD40, CD86 and CD163 expression).
